# Enhancing surgical precision: unveiling the impact of preoperative colonoscopy in anal fistula patients

**DOI:** 10.1186/s12876-023-03066-x

**Published:** 2023-12-15

**Authors:** Shumin Xu, Luo Zhang, Zhi Li, Kaiping Wang, Fang Liu, Bo Cao

**Affiliations:** 1 Department of Anorectal Surgery, Guizhou Province, The First Affiliated Hospital of Guizhou University of Traditional Chinese Medicine, No. 71, Baoshan North Road, Guiyang, 550000 P. R. China; 2 Dental Department, Guizhou Provincial People’s Hospital, Guiyang, 550000 P. R. China

**Keywords:** Anal diseases, Anal fistula, Preoperative examination, Colonoscopy, Retrospective analysis

## Abstract

**Background:**

Anal fistula is a common benign anorectal disease that often requires surgical intervention for effective treatment. In recent years, preoperative colonoscopy as a diagnostic tool in patients with anal fistula has garnered increasing attention due to its potential clinical application value. By investigating underlying inflammatory bowel disease (IBD), polyps, and other abnormalities, preoperative colonoscopy can offer insights to refine surgical strategies and improve patient outcomes.

**Methods:**

This retrospective study focused on 1796 patients with various benign anorectal diseases who underwent preoperative intestinal endoscopy and met surgical criteria within the preceding three years at the First Affiliated Hospital of Guizhou University of Traditional Chinese Medicine. Among these patients, 949 diagnosed with anal fistula comprised group A, while 847 patients without anal fistula were assigned to group B for comparison. The investigation encompassed an analysis of general patient information, endoscopic findings, polyp histopathology, distribution of bowel inflammation sites, and results of inflammatory bowel disease assessments between the two patient cohorts. A subgroup analysis was also conducted on 2275 anal fistula patients with no surgical contraindications. This subgroup was categorized into Group A (949 patients who underwent preoperative intestinal endoscopy) and Group C (1326 patients who did not undergo preoperative colonoscopy). The study compared the rates of detecting endoscopic lesions and IBD-related findings between the two subgroups.

**Results:**

The study initially confirmed the comparability of general patient information between groups A and B. Notably, the abnormal detection rate in group A was significantly higher than in group B (*P* < 0.01). In terms of endoscopic findings, the anal fistula group (group A) exhibited higher rates of detecting bowel inflammation, inflammatory bowel disease, and polyps compared to the non-anal fistula group (group B) (*P* < 0.05). The distribution of inflammation locations indicated higher detection rates in the terminal ileum, ileocecal region, and ascending colon for group A compared to group B (*P* < 0.05). Although the incidence of IBD in group A was higher than in group B, this difference did not reach statistical significance (*P* > 0.05). Subsequently, the analysis of the subgroup (groups A and C) revealed a significant disparity in intestinal endoscopic detection rates (*P* < 0.01) and statistically significant differences in detecting IBD (*P* < 0.05) and Crohn's disease (*P* < 0.05) between the two anal fistula subgroups.

**Conclusions:**

The findings of this study underscore the substantial clinical value of preoperative colonoscopy in the comprehensive evaluation of patients with anal fistula. Preoperative colonoscopy aids in ruling out localized perianal lesions caused by underlying inflammatory bowel disease, thereby mitigating the likelihood of missed diagnoses and enhancing treatment outcomes. This research highlights the importance of incorporating preoperative colonoscopy as a valuable diagnostic tool in managing anal fistula patients.

## Introduction

An anal fistula is a common condition in proctology, often presenting symptoms such as discomfort, recurrent abscesses or ulcers, discharge of fluid and pus, or anal itching [1]. In China, the estimated incidence of anal fistula among anorectal diseases ranges between 1.67% to 3.6%, predominantly affecting younger individuals. Males are observed to have a higher prevalence than females [2]. While surgical intervention remains the mainstay of treatment for anal fistula, many patients experience prolonged post-operative healing times and elevated recurrence rates [3–5]. Recent research has suggested that the risk of recurrence post-anal fistula surgery can be as high as 57%, with one major contributing factor being concurrent intestinal pathologies such as inflammatory bowel disease (IBD) and intestinal tuberculosis [6]. This subset of anal fistulas, characterized by their complexity, surgical challenges, and susceptibility to recurrence, presents a notable challenge in proctology [7]. Regrettably, the absence of distinct intestinal pathology manifestations has resulted in a high rate of underdiagnosis of these conditions [8]. Precise preoperative diagnosis and assessment are pivotal to enhancing surgical accuracy and reducing recurrence [9].

In recent years, the use of preoperative colonoscopy in patients with anal fistula has seen an uptick due to its potential clinical applicability [10]. Preoperative colonoscopy presents an opportunity to identify conditions like ulcerative colitis (UC), Crohn's disease (CD), isolated ileocecal inflammation, intestinal tuberculosis, and gastrointestinal tumors, thereby facilitating the refinement of treatment plans and prognosis determination [11]. Such concomitant conditions can influence treatment strategies and lead to postoperative recurrence [12].

Preoperative colonoscopy can uncover potential underlying causes behind an anal fistula, such as inflammatory bowel disease or polyps, which might affect surgical options and treatment strategies [13]. Beyond assessing the overall health of the intestines, colonoscopy can offer invaluable insights regarding the complexity of the fistula and potential complications, enabling the clinician to devise a more accurate treatment plan [14]. Thus, the clinical application of preoperative colonoscopy holds significant importance for accurate surgical diagnosis, treatment and ensuring medical safety [15].

However, currently, there is a lack of sufficient clinical trial evidence to demonstrate the statistical significance and benefits of preoperative endoscopic examination in determining the gastrointestinal health status, assisting in the precise formulation of surgical plans, and predicting postoperative outcomes for patients. This hinders the comprehensive application of preoperative colonoscopy in the diagnosis and treatment of anal fistula patients. In order to address these clinical challenges and improve the value of preoperative colonoscopy, this study conducted endoscopic examinations on 1796 patients with benign anorectal diseases. These patients were categorized into anal fistula and non-anal fistula groups. By contrasting the colonoscopic examination outcomes between these two groups, assessing lesion detection rates, locations of inflammatory lesions, polyp characteristics, and incidence of inflammatory bowel diseases, we aimed to elucidate the clinical diagnostic value of preoperative endoscopy in patients with anal fistulas. Furthermore, to delve deeper into the necessity of preoperative colonoscopy for patients with anal fistula, an additional cohort of 2275 patients with anal fistula was incorporated into this study. They were segregated into two groups based on whether they underwent preoperative colonoscopy. A retrospective comparison was made between the two subgroups of anal fistula patients: those diagnosed with intestinal pathology post-preoperative colonoscopy and those who manifested intestinal pathology due to unfavorable prognosis without preoperative colonoscopy. This comparative analysis seeks to furnish insights for clinicians in diagnosing and treating patients with anal fistulas. Subsequent sections will delineate the findings of this research.

## Materials and methods

### Ethical statement

The study obtained ethical approval from the Institutional Review Board of the First Affiliated Hospital of Guizhou University of Traditional Chinese Medicine. Due to the retrospective design of the investigation, the ethics committee waived the need for individual informed consent. All research procedures strictly followed the principles outlined in the Declaration of Helsinki and complied with the guidelines and regulations of our institution.

### Study design and participants

**Fig. 1:**
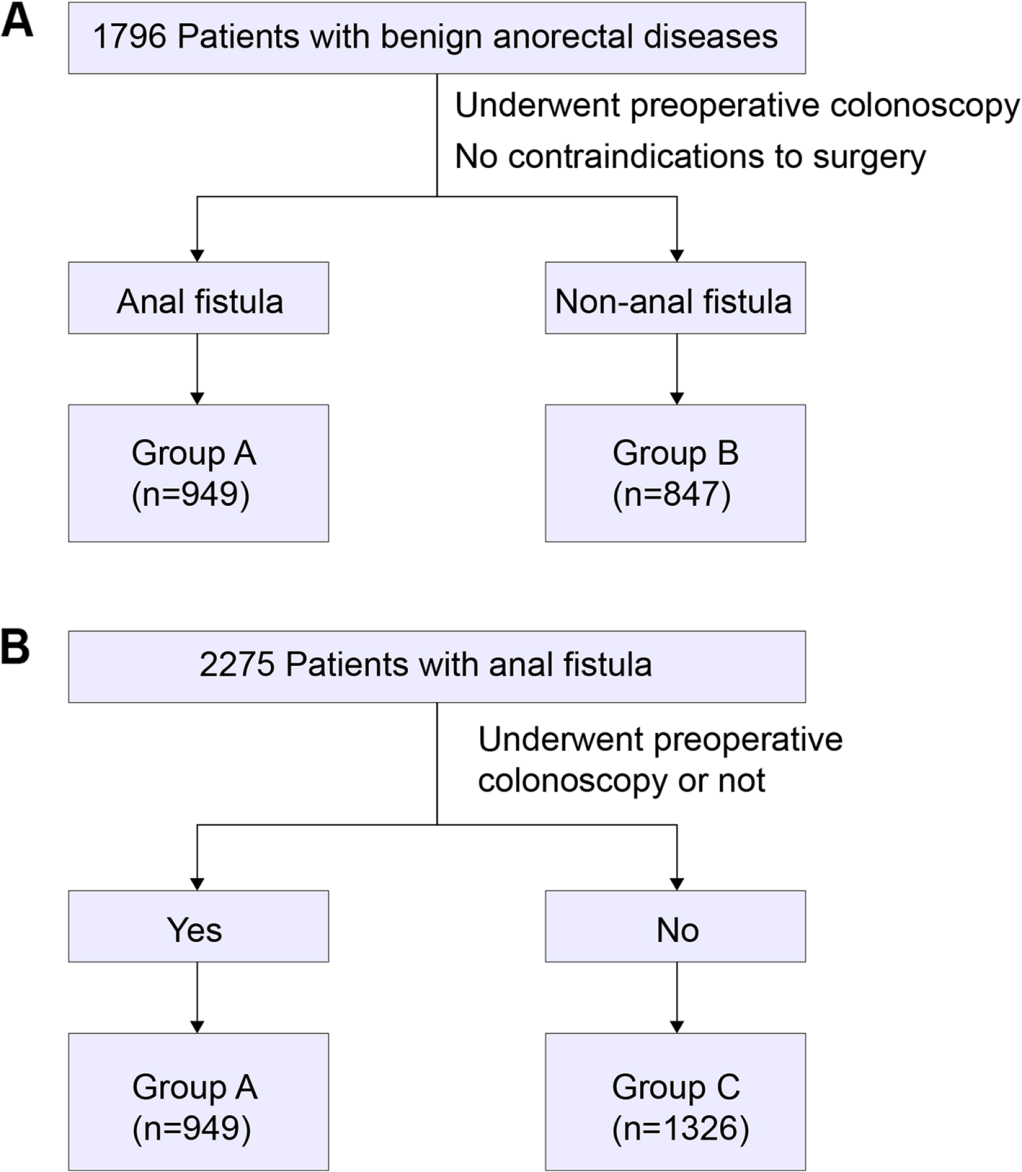
Patient grouping process in the clinical study. Note: **A** The schematic diagram of the grouping process for Groups A and B, patients diagnosed with anal fistula are placed in Group A, while patients without anal fistula are placed in Group B; **B** The schematic diagram of the grouping process for Groups A and C, patients who have undergone preoperative colonoscopy are placed in Group A, while patients who have not undergone the examination are placed in Group C

This research adopted a retrospective cohort study design, targeting the evaluation of the clinical relevance of preoperative colonoscopy in anal fistula patients. The study was anchored at the First Affiliated Hospital of Guizhou University of Traditional Chinese Medicine and spanned three years. From the initial pool, 1796 patients diagnosed with various benign anorectal diseases and who underwent preoperative intestinal endoscopy within this duration were enrolled. These participants were further segmented into two primary cohorts: Group A, composed of 949 patients diagnosed with anal fistula and Group B, consisting of 847 patients without anal fistula, serving as the comparative cohort. An auxiliary subgroup analysis encompassed 2275 anal fistula patients, ensuring that these participants lacked surgical contraindications. Within this subgroup, Group A housed 949 patients who underwent preoperative intestinal endoscopy, while Group C included 1326 patients without prior preoperative colonoscopy. The patient screening process is illustrated in Fig. 1.

Eligibility for this study demanded that patients were between 18 and 75 years old, had a clinical diagnosis of benign anorectal disease, underwent preoperative intestinal endoscopy in the past three years, and consented for their anonymized data to be used for research. Moreover, accessible and comprehensive medical documentation was a prerequisite. On the contrary, patients were excluded if they had a history of malignant anorectal diseases, known inflammatory bowel disease before the study, had prior surgical interventions for anorectal diseases before the study's onset, were pregnant or breastfeeding, had other severe systemic ailments like autoimmune diseases or chronic infections, or held incomplete medical records. Additionally, those contraindicated for endoscopic procedures, mainly due to severe cardiac or pulmonary diseases, were left out.

Data extraction ensued upon classifying patients based on these criteria, focusing on general patient details, endoscopic observations, polyp histopathological outcomes, bowel inflammation site distributions, and inflammatory bowel disease evaluation results. Data was meticulously extracted from hospital archives, ensuring that the sanctity of patient confidentiality remained uncompromised. This streamlined methodological structure ensures the study's integrity while highlighting the nuanced implications of preoperative colonoscopy in anal fistula management.

### Preoperative procedure

Per the Chinese bowel preparation guidelines [16], patients from both cohorts were mandated to adhere to a low-fiber dietary regimen commencing a day before the scheduled examination. Specifically, patients were directed to abstain from food and water consumption post-22:00 h on the day preceding the examination. This fasting duration was suitably extended if any were identified with gastrointestinal motility disorders. As a part of the bowel cleansing protocol, they were administered an oral dose of laxative, specifically polyethylene glycol electrolyte dispersion (68.56 g/bag), under physician guidance. I culminated in a defecation phase characterized by a clear yellow, watery stool devoid of Any residual fecal remnants. Concluding the examination, patients maintained their fasting regimen, restricting their food and water intake for 2 h. Subsequently, they were provided with a liquid soft diet for the remainder of the day.

### Outcome measures

In this study, the primary outcome measures were derived from the results of the preoperative colonoscopies. Specifically, any abnormalities detected during the procedure, ranging from inflammation to polyps, tumors, and other abnormal growths or lesions, were meticulously recorded. Moreover, when enterocolitis lesions were identified, their specific locations within the gastrointestinal tract were documented to provide insights into potential disease patterns and inform therapeutic strategies. Crucially, all lesion tissues observed during the colonoscopy, including those indicative of polyps, inflammatory bowel disease, or tumors, were subjected to either excision or biopsy for subsequent pathological assessment. This in-depth evaluation allowed for a comparative analysis of detection rates, intrinsic nature, and distinct pathological characteristics among the patient cohorts, ensuring a holistic understanding of the underlying abnormalities and their implications for anal fistula management.

### Statistical analysis

In this study, data analysis employed a myriad of statistical techniques. Demographics and baseline patient information were expressed using descriptive statistics, with continuous variables like age presented as means ± SDs and compared through the independent t-test, while categorical variables were expressed as frequencies and percentages and analyzed using Chi-square or Fisher's exact tests. Endoscopic findings were compared using the Chi-square test, including rates of detecting bowel inflammation, IBD, and polyps between groups. Furthermore, specific locations of bowel inflammation (terminal ileum, ileocecal region, and ascending colon) in groups A and B were statistically analyzed. A subgroup analysis focused on the anal fistula patients' endoscopic lesion detection rates, IBD-related findings, and Crohn's disease occurrences, contrasting those who underwent preoperative intestinal endoscopy and those who didn't. Multivariate logistic regression was adjusted for potential confounders and pinpointed factors significantly tied to lesion occurrences and IBD detection rates. The diagnostic utility of preoperative colonoscopy was evaluated by calculating its sensitivity, specificity, positive predictive value, and negative predictive value. For all tests, a two-tailed *P*-value less than 0.05 denoted statistical significance. Analyses were facilitated using SPSS software (version 25.0, IBM Corp, Armonk, NY, USA) with graphical visuals generated via GraphPad Prism 8. The data rigorously adhered to all underlying statistical test assumptions.

## Results

### Deciphering patient profiles and unveiling key differences in endoscopic outcomes between groups

**Fig. 2:**
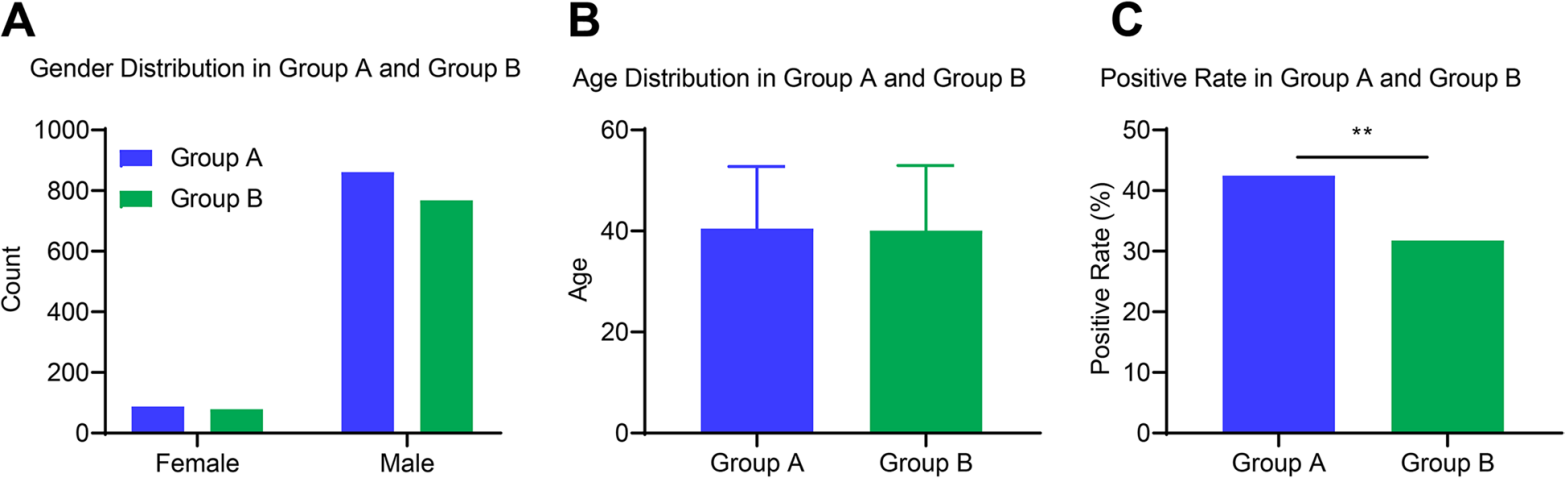
Demographics and endoscopic findings in groups a and b. Note: **A** The bar chart displays the gender distribution of Group A and Group B; **B** The bar chart shows the distribution of average age and standard deviation for Group A and Group B; **C** The bar chart represents the positive detection rate of intestinal lesions in Group A and Group B. Group A (blue, *n* = 949), Group B (green, *n* = 847), **P < 0.001

Let's delve into the specifics to provide a comprehensive understanding of the patient demographics and their endoscopic findings. In Fig. 2, Group A comprised 88 females and 861 males, while Group B comprised 79 females and 768 males. Although there were slightly more males and females in Group A than in Group B, the gender distribution between the two groups did not show a statistically significant difference (*P* = 0.969) (Fig. 2A). The average age of patients in Group A was 40.47 years with a standard deviation of 12.28 years; in Group B, it was 40.08 years with a standard deviation of 12.89 years. Despite the slight differences in average age, the variations were not statistically significant (*P* = 0.985) (Fig. 2B). Notably, 42.47% of patients in Group A were detected with lesions during the intestinal endoscopy, as opposed to 31.76% in Group B. This result indicates that the detection rate of lesions in Group A was significantly higher than in Group B, with the difference being statistically significant (*P* < 0.001) (Fig. 2C). In summary, while the demographics between the two groups were comparable, the endoscopic findings highlighted a pronounced difference in the lesion detection rate, favoring Group A.

### Comparative analysis of lesion detection rates in intestinal endoscopic examinations: group a vs. group b insights

**Fig. 3:**
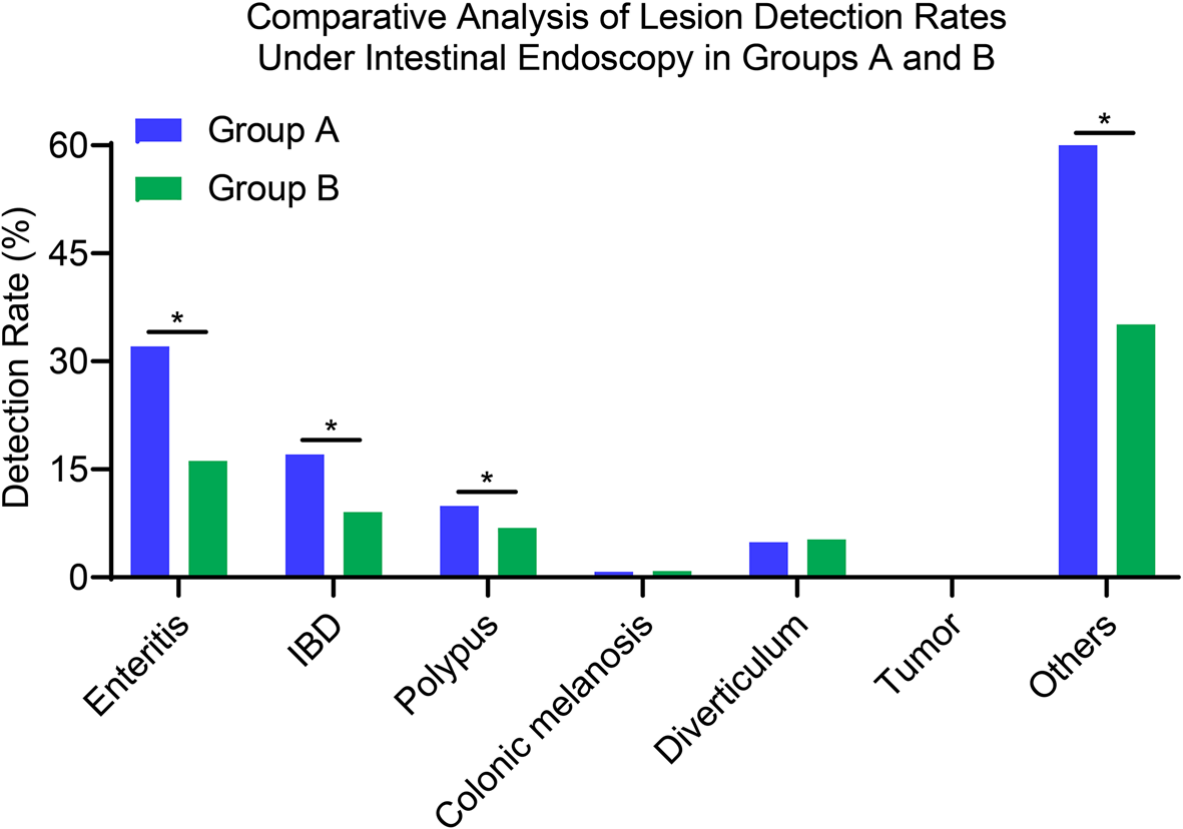
Detection rates of intestinal lesions: a comparative study between groups a and b. Note: Categories include enteritis, inflammatory bowel disease, polyps, colonic melanosis, diverticula, tumors, and other conditions, *P < 0.05

Diving deeper into the lesion findings from the intestinal endoscopic examinations, a number of insights emerge from Fig. 3. In Group A, the detection rates for conditions such as Enteritis (32.09%), Inflammatory Bowel Disease (IBD) (17.06%), and Polyps (9.94%) were notably higher than those in Group B, which stood at 16.18%, 9.06%, and 6.88%, respectively. Conversely, for conditions like Colonic Melanosis, Diverticulum, and Tumor, the detection rates between the two groups were relatively close, with Group A recording rates of 0.79%, 4.89%, and 0.10% against Group B's 0.88%, 5.29%, and 0.15%, respectively. A stark contrast was observed in the 'Others' category, where Group B's detection rate of 61.47% significantly outstripped Group A's 35.13%. In summation, while there were distinct variations in the detection rates of certain conditions between the two groups, both groups presented a mix of higher and comparable rates across different lesions.

### Unveiling polyp pathways: a comparative analysis of pathological diagnoses across two groups

**Fig. 4:**
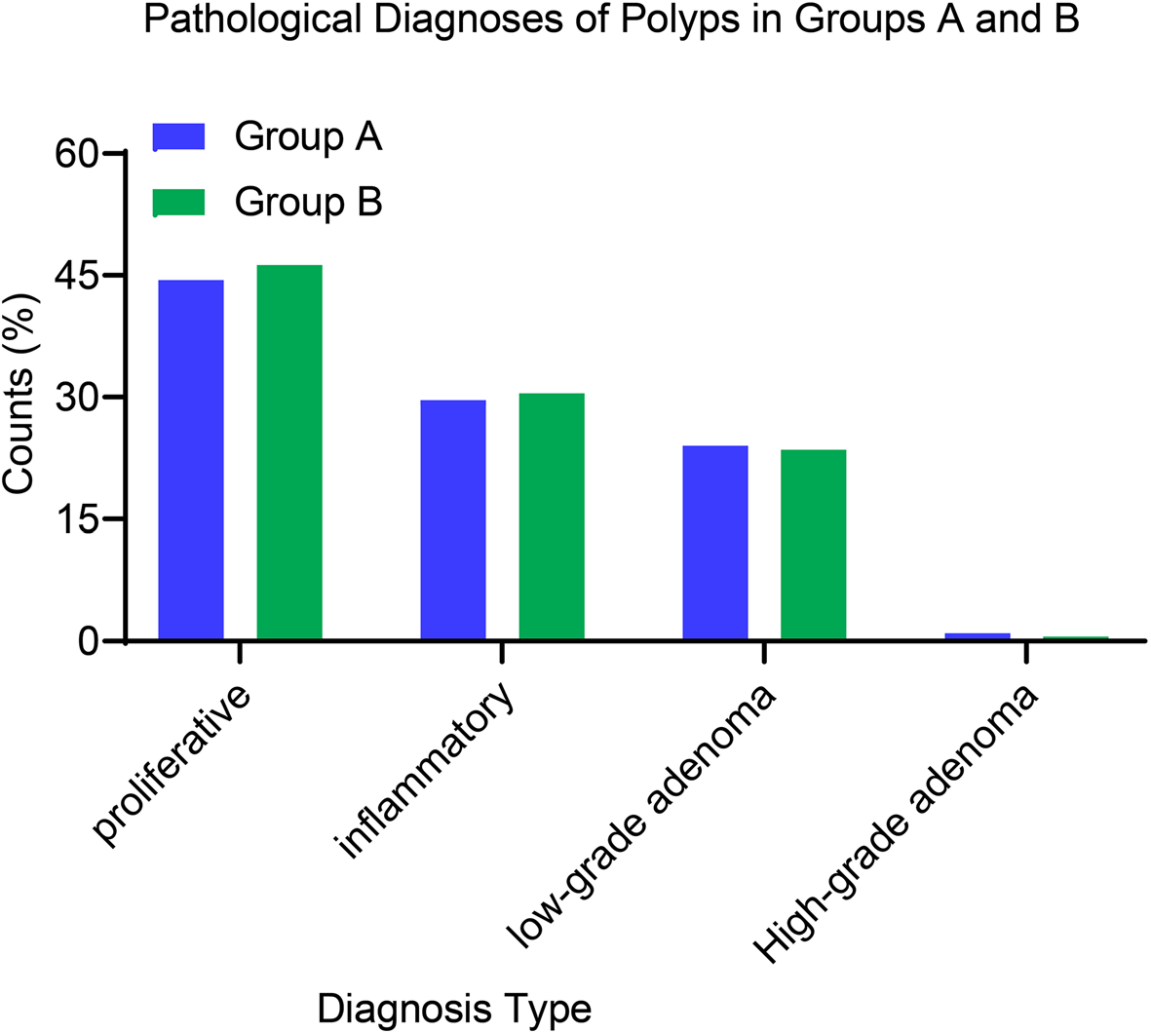
Pathological diagnoses of polyps: group A vs. Group B Insights. Note: The classification includes proliferative, inflammatory, low-grade adenoma, and high-grade adenoma

Through a meticulous exploration of the subtle differences in the pathological diagnosis of polyps between two groups, several relevant observations were uncovered. In terms of proliferative diagnoses, Group A reported 228 instances, accounting for 44.44%, closely followed by Group B with 236 instances, making up 46.27%. As for inflammatory diagnoses, the situations in Group A and Group B were nearly equal, with 152 cases (29.63%) and 155 cases (30.45%) respectively. In the case of low-grade adenoma, Group A had 123 diagnoses (24%), while Group B had a similar situation with 120 cases, accounting for 23.53%. High-grade adenoma diagnoses were infrequent in both groups, with 5 instances (0.98%) in Group A and 3 instances (0.59%) in Group B (Fig. 4). The overall data strongly emphasizes that there are notable similarities in the pathological diagnosis of polyps between the two groups, and they do not exhibit statistically significant differences.

### Enteritis unveiled: unearthing detection discrepancies between groups A and B

**Fig. 5:**
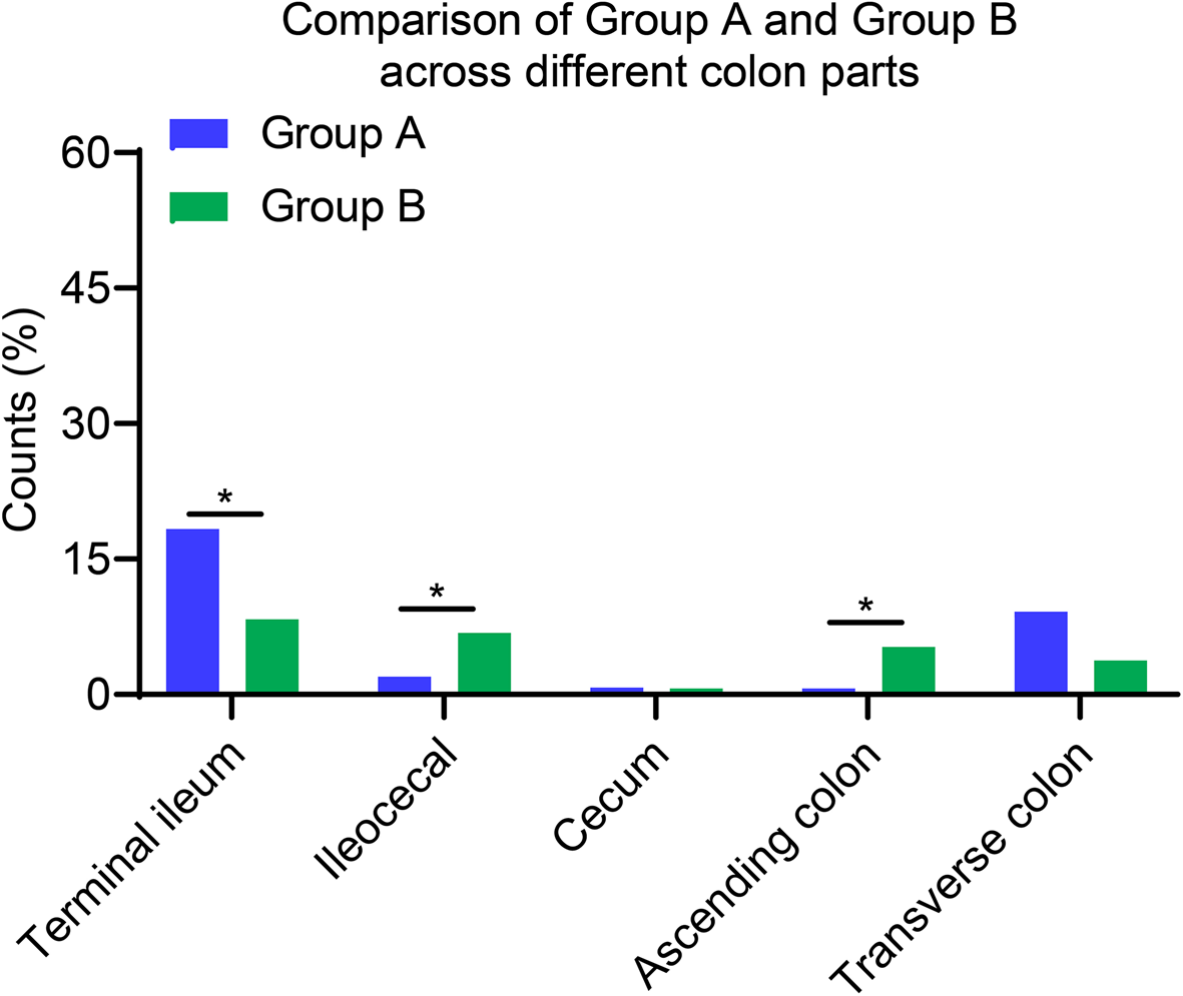
Distribution of enteritis across gastrointestinal sites: a comparative View of Groups A and B. Note: The classifications include terminal ileum, ileocecal region, cecum, ascending colon, transverse colon, descending colon. *P < 0.05

We delved into the distribution of enteritis across various colonic regions among two groups. At the Terminal ileum, Group A exhibited a detection rate of 18.30%, notably surpassing Group B's rate of 8.33%, a statistically significant difference (*P* < 0.05). The Ileocecal region showed a similar trend, albeit with Group B having a slightly higher detection rate of 6.82% compared to Group A's 1.96%, and this variance was also statistically significant. Both groups had a minimal presence of enteritis in the Cecum, with detection rates of 0.76% for Group A and 0.65% for Group B, and no significant difference was observed statistically. In the Ascending colon, although Group B's detection rate of 5.30% was higher than Group A's 0.65%, the discrepancy between the groups was statistically significant. However, the difference did not reach statistical significance for the Transverse colon, where Group A had a rate of 9.15% compared to Group B's 3.79% (Fig. 5). In summary, while Group A displayed a higher prevalence of enteritis at certain colonic sites, not all differences between the groups were statistically significant.

### Comparative prevalence of ulcerative colitis and crohn's disease in groups A and B: A statistical insight

**Fig. 6:**
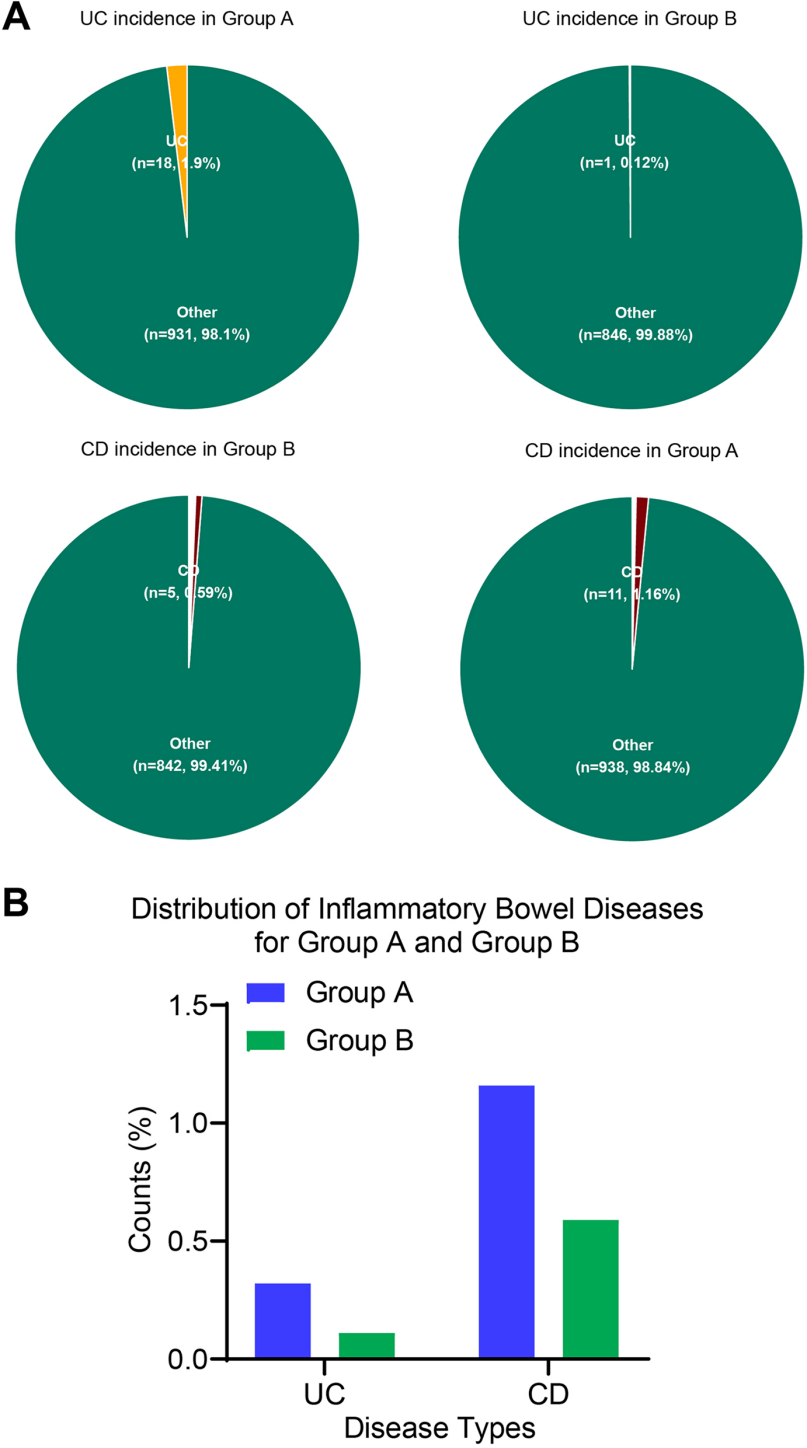
Prevalence of IBD in Groups A and B: A detailed examination. Note: **A** Presenting pie charts of UC and CD incidence rates in Group A and Group B; **B** Bar chart depicting the number of UC and CD cases in Group A and Group B, with blue representing Group A and green representing Group B. *P < 0.05

Diving deeper into the data presented in Fig. 6 offers a comprehensive perspective on the prevalence of inflammatory bowel diseases across two distinct groups. In our meticulous examination, we observed that Group A recorded a prevalence of 0.32% for Ulcerative Colitis (UC), representing 18 cases out of 949 participants. In contrast, Group B showcased a slightly lower prevalence of 0.11%, with just 1 case among 847 participants. Turning our attention to Crohn's Disease (CD), Group A reported a prevalence of 1.16%, equivalent to 11 cases from their cohort. Group B's prevalence was 0.59%, corresponding to 5 cases within their participant base. While the data reveals Group A having a marginally higher prevalence in both UC and CD compared to Group B, statistical inferences confirm the absence of any significant disparity in IBD distribution between the groups (*P* > 0.05). The two groups present closely aligned IBD distributions, with no significant deviations underlined by statistical evaluations.

### Differential positive rates in colonoscopy examinations: a comparative analysis between group A and group C

**Fig. 7:**
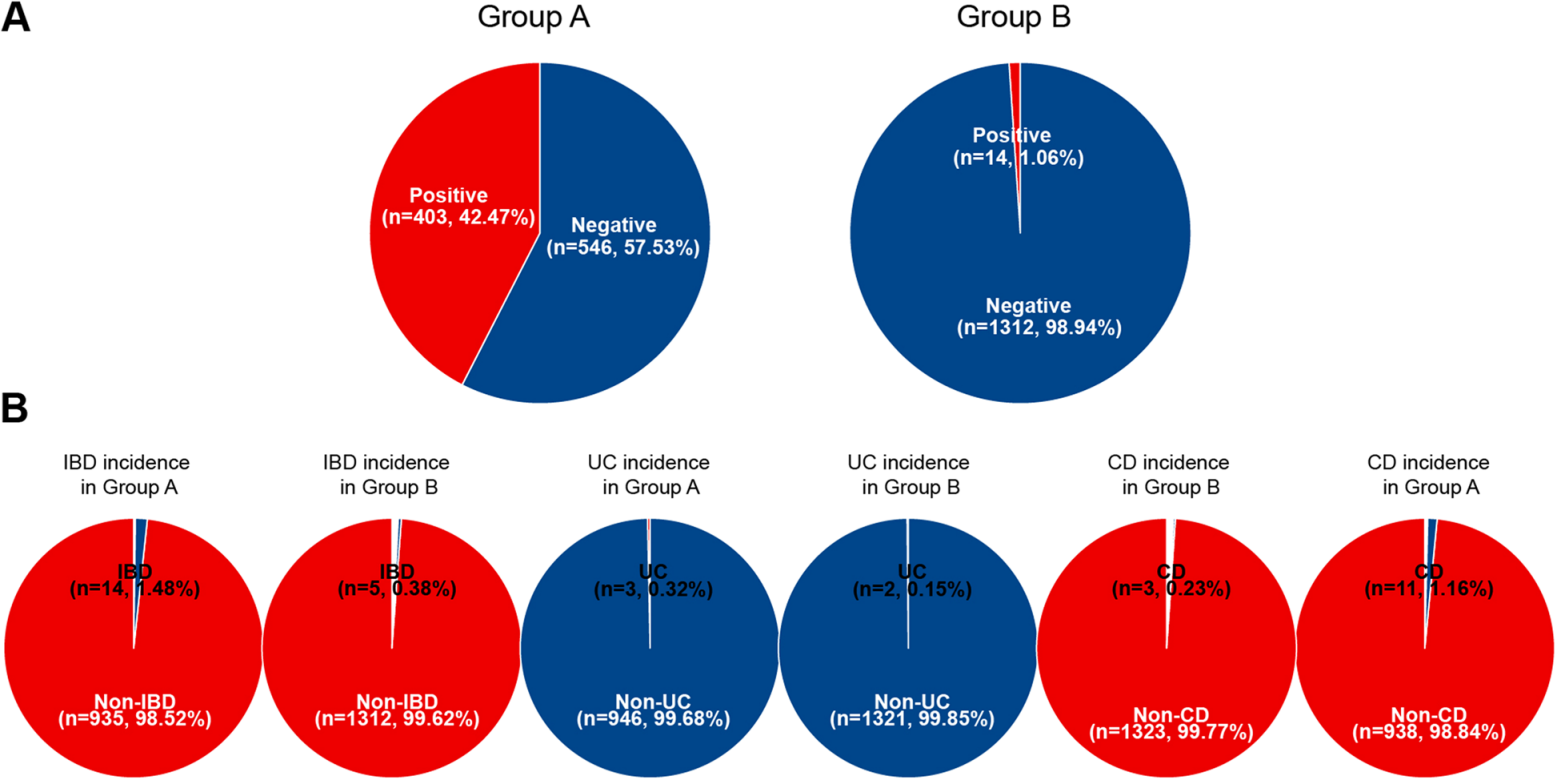
Comparison of disease incidence between preoperative examined and non-examined anal fistula groups: A vs. C. Note: **A** Pie chart illustrating the overall positive rate in Group A and Group C; **B **Pie chart showing IBD, UC, and CD incidence rates in Group A and Group C

Preoperative colonoscopy examination in anal fistula patients garners increasing attention in clinical applications, especially in diagnostic and treatment planning. Addressing this, we meticulously analyzed the data from Fig. 7. Comparing the positive rates between Group A and Group C, we found that Group A's overall positive rate stood at 42.47%, significantly higher than Group C's 1.06% (*P* < 0.05) (Fig. 7A). Delving deeper into the positive rates for inflammatory bowel disease (IBD), ulcerative colitis (UC), and Crohn's disease (CD), the results revealed that Group A's positive rates for IBD and CD were 1.48% and 1.16%, respectively, both notably higher than Group C's rates of 0.38% and 0.23% (*P* < 0.05) (Fig. 7B). However, regarding the UC positive rate, no statistically significant difference was observed between the two groups, with Group A at 0.32% and Group C at 0.15% (Fig. 7B). These findings strongly suggest that, before undergoing anal fistula surgery, a colonoscopy examination can be a valuable diagnostic tool, aiding in a more accurate evaluation and diagnosis of patients, thereby optimizing treatment plans.

## Discussion

In recent years, colonoscopy has become crucial for screening and monitoring gastrointestinal tumors and assessing benign anorectal diseases [17]. In this context, anal fistula is a prevalent anorectal disease characterized by complex presentations such as multiple fistulas, intricate tracts, or persistent non-healing after multiple surgeries, often accompanied by high recurrence rates [18]. Given this complex nature, considering the possibility of concurrent inflammatory bowel disease, intestinal tuberculosis, and other pathologies is essential [19]. Hence, preoperative colonoscopy provides a valuable means to elucidate the patients' intestinal conditions [20].

Our study consistently demonstrated a relatively high overall abnormal detection rate for preoperative colonoscopy, with 42.47% in anal fistula patients and 31.76% in non-anal fistula patients. Combining the statistical analysis of anal fistula patients who underwent preoperative colonoscopy with those who did not, the study found that preoperative colonoscopy holds significant value in assessing the disease status of anal fistula patients and warrants careful consideration.

The colonoscopic findings of this study emphasized that enteritis, inflammatory bowel disease, and polyps were the top three pathologies [21]. Perianal involvement of Crohn's disease (CD) has garnered increasing attention, with anal fistulas being a common manifestation [22–24]. The reported prevalence of CD-related perianal fistulas is around 43% [22], and the occurrence rates of anal fistulas within 1 year, 10 years, and 20 years of celiac disease diagnosis are 12%, 21%, and 26%, respectively [25]. Single surgical interventions often lead to high recurrence and reoperation rates for patients, significantly impacting their quality of life [26]. Our study results indicated that the detection rates of small bowel and colonic inflammation and inflammatory bowel disease were higher in the anal fistula group compared to the non-anal fistula group. Notably, the anal fistula group showed significantly higher detection rates of small bowel and colonic inflammation in the terminal ileum, ileocecal area, and ascending colon. It underscores the need to carefully consider anal fistulas associated with enteritis and inflammatory bowel disease, particularly regarding terminal ileum enteritis, which might represent a latent stage of celiac disease.

Furthermore, the prolonged recovery time after anal fistula surgery often hinders timely postoperative follow-up, leading to missed opportunities for early treatment [27]. It could adversely affect disease prognosis, increase treatment risks and financial burden, and even result in medical disputes [28]. By revealing the higher detection rate of inflammatory bowel disease in anal fistula patients, our study reinforces the benefits of preoperative colonoscopy. Therefore, preoperative colonoscopy contributes to formulating informed and prudent treatment strategies.

Against the backdrop of the increasing incidence of colorectal polyps, understanding the clinical and epidemiological characteristics is significant in colorectal cancer prevention [29]. Early detection and removal of precancerous lesions such as adenomatous polyps effectively inhibit disease progression and deterioration [30]. Prior research indicates that early removal of colon polyps can reduce the incidence of colorectal cancer by up to 30% [31]. Moreover, malignancies are sometimes only discovered postoperatively for benign anorectal diseases, with some diagnosed at advanced stages [32]. Our study's findings support these notions, with a relatively high detection rate of polyps in the dataset, with nearly 65% of polyps being adenomas based on pathological examination. It underscores that preoperative colonoscopy for anorectal diseases enables comprehensive evaluation for anal fistula patients for holistic medical and surgical management and aids in the early detection and treatment of precursor lesions, significantly reducing the risk of tumors.

**Fig. 8:**
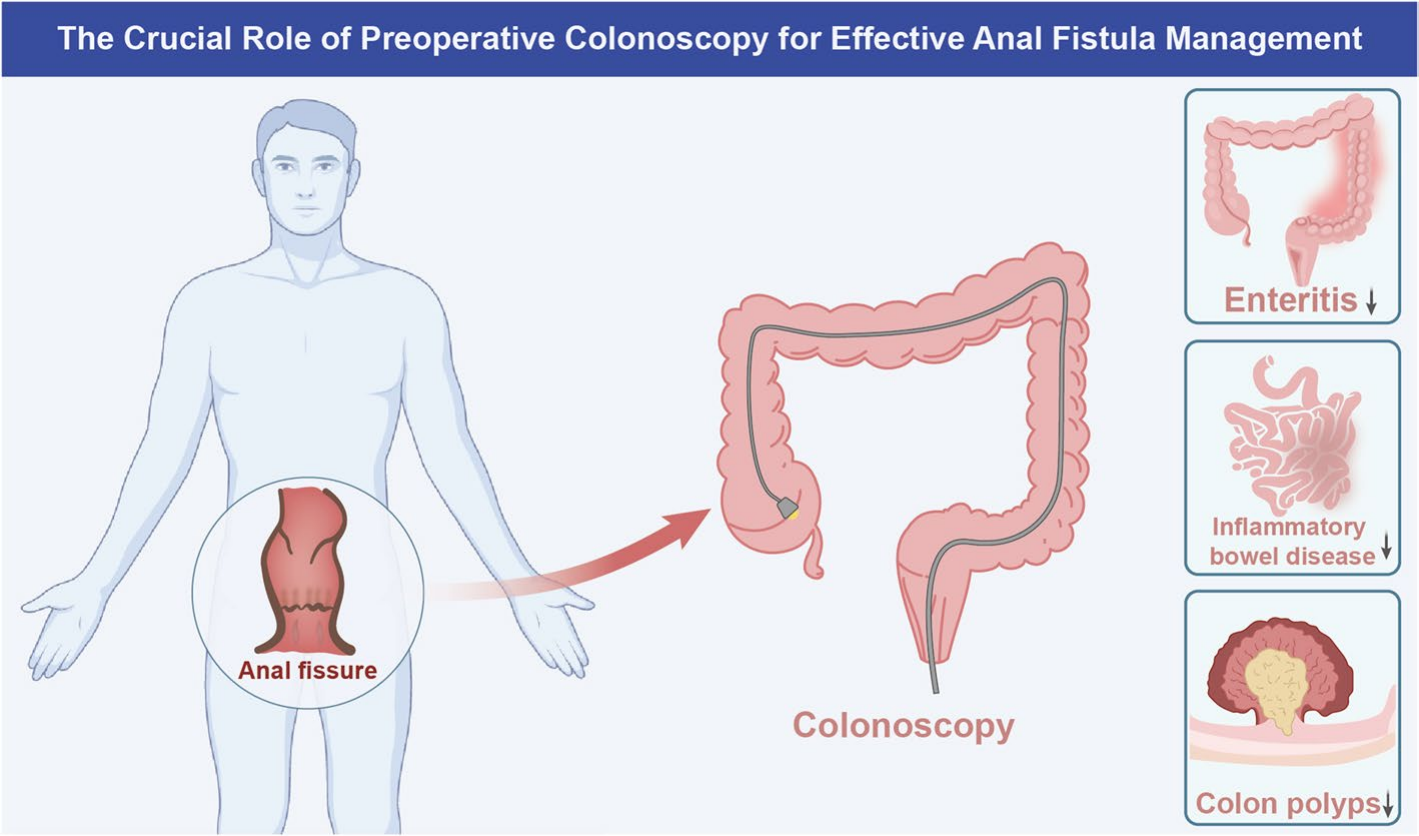
Visualizing the impact of preoperative colonoscopy on surgical strategy for anal fistula patients

In conclusion, the close association between anal fistula development and the inflammatory environment of the intestine is evident in the higher likelihood of patients with anal fistula developing inflammatory bowel diseases and related symptoms such as enteritis, inflammatory bowel disease, and polyps. Active preoperative colonoscopy can significantly mitigate the risk of missed diagnoses in anal fistula patients, leading to enhanced cure rates, reduced recurrence rates, and alleviating patient discomfort through integrated medical and surgical treatments (Fig. 8). As such, preoperative colonoscopy transcends being merely a tool for colorectal cancer screening; it becomes an imperative investigation for patients with anorectal diseases.

In prospect, the insights garnered from this study lay the groundwork for a broader understanding of the complex interplay between anal fistula and inflammatory bowel diseases. Future research can expand the dataset and refine survey methodologies, leading to refined insights that offer more comprehensive guidance for healthcare practitioners in managing this challenging condition.

## Limitations

Although our study highlights the significant importance of preoperative endoscopic examination in the clinical diagnosis and treatment of anal fistula patients, there are still limitations that hinder the rapid dissemination of our findings in the clinical setting. Firstly, there is a limitation in the collection of our samples due to the fact that this survey was based on a single-center, three-year dataset, resulting in a small sample size. In future research, we intend to include multi-center, long-term patient samples to further validate our findings in a larger population. Additionally, this study only considers inflammatory bowel disease as a contributing factor, but in subsequent studies, we aim to broaden the scope of our research by incorporating more investigative parameters to obtain comprehensive data on the correlation between colonoscopy findings and anal fissure disease. This will enable us to guide more accurate clinical diagnosis and treatment strategies.

## Data Availability

The datasets used and/or analyzed during the current study are available from the corresponding author upon reasonable request.

## Abbreviations

IBD: Inflammatory bowel disease
UC: Ulcerative colitis
CD: Crohn's disease
